# Research in child and adolescent psychiatry in India

**DOI:** 10.4103/0019-5545.69235

**Published:** 2010-01

**Authors:** Priyavadan Chandrakant Shastri, Jay P. Shastri, Dimple Shastri

**Affiliations:** Department of Psychiatry, B.Y.L. Nair Hospital and T.N. Medical College, Mumbai, India; 1 Sir Kikabhai Premchand’s MIND’s College of Special Education, Mumbai, India; 2 Shrimati Motibai Thakersey Institute for Research in field of mental retardation, Child Development Center, Mumbai, India

**Keywords:** Child, adolescent, mental health, mental illness, original articles, review articles, case reports

## Abstract

The primary source for this annotation on child and adolescent psychiatry is Indian Journal of Psychiatry. Articles covering various dimensions of child and adolescent mental health were searched from its electronic data base to discuss relevant articles. Literature was mainly in the form of original research articles, review articles, case reports, editorials, orations and presidential address.

## INTRODUCTION

In India, child mental health services have been neglected in the last 63 years. National Mental Health policy makers (2003) have practically nothing on their agenda as far as child mental health policy and planning are concerned. It is a sorry state of affairs. In last 67 years, from 1937 when first child guidance clinic was introduced till 2003, NIPCCD study located only 164 Child Guidance Cinics (CGC); roughly two CGC a year and that too only in metro cities and mega cities. All these child guidance movements and mental health activities, services have been initiated and sustained by efforts from non-governmental organizations (NGOs).[1]

Ten per cent of the child population is in need of special care and treatment. Only one out of the 100 gets some care and treatment. It is high time we reach out to 99% of the child population that is being unattended by any agency. Children with borderline intellectual functioning and various learning, speech, visual and hearing difficulties are conservatively estimated to be 20% of the total child population. These 114 million children have no facilities even in the urban areas.[1]

Child population is not homogeneous. Large numbers of children have no home, school and family. They can be in orphanages, destitute homes, beggars’ homes, juvenile homes, rescue homes and remand homes. They can even be street children. All these groups some how have their own self-help group, one of the motto of such group is “each one teach one” to become self-sufficient. Some of them run their own child guidance clinic.[1]

Child psychiatry in the West expanded rapidly in the 60s and 70s. Strikingly, Indian Child Psychiatry has been part of general psychiatrist and that trend has grown much more in last two decades. Mumbai, Bangalore and Lucknow had considerable child mental health activity. General psychiatrist offered their services to children in many diverse ways. They are to be found working in many fields child’s home, remand home, rescue home, delinquency, cerebral palsy and other handicap.[2]

### Multidisciplinary child practice

Child mental health is not in the province of child psychiatry alone. Education, social welfare, primary health services, even community leaders and neighbors contribute more than child psychiatry on its own. The team work always does wonders in child psychiatry. Important key members of the team include psychiatrist, psychologist, social worker, neurologist, pediatrician, occupational therapist, speech therapist, physiotherapist, special educator, art, music, drama therapist and cytogenetic expert.[2]

### Cooperation with the educational services

School Mental Health has been a major mental health movement which covers up the large population of children and adolescent, but has been effectively implemented only in metros and not in smaller towns and urban areas in the last four decades. Research publications during the 60s, 70s and 80s reported that mental retardation formed bulk of population attending CGC during that period. While emotional and behavioral disturbances were less identified and referred. The trend has changed. All spectrums of diagnostic categories are now referred and treated at various teaching hospitals, psychiatry departments, pediatric departments, various colleges of social work and large number run by NGOs.[3]

The last three decades has shown highly specialized clinics rendering specialized services to children with learning disability, autism, cerebral palsy and mental retardation mostly in metros and urban areas. Such centers do run the genetic clinic and research in specific disorders. Development clinics for 0 to 3 years age group for various disability groups and multiple disability groups have special focus of identification, assessment and therapy. All these centers are attended by general psychiatrist rendering highly specialized services.[3]

Child Mental Health Policy and School Mental Health programs have provided excellent opportunity to enhance mental health program for children and adolescents. The focus is rightly on preschool children and school based mental health program, which will prevent illness and possibly promote positive mental health. It also ensures that it will reduce behavior disorders in children and prevent adult psychopathology. Effectiveness of child mental health intervention programs will surely help in addressing mental health disorders among adults.[4]

In order to achieve desired outcomes one should embrace all those services that contribute to the mental health care of children and adolescents, whether provided by health, education, social services or other agencies. It is also crucial to partner with services whose primary function is not mental health care, such as GPs and schools. They can always contribute by offering general advice and treatment for less severe problems, contribute towards mental health promotion, identify problems early in their development and refer to more specialist services. This is to explicitly acknowledge that supporting children and adolescent with mental health problems is not the responsibility of specialist services alone.[4] Importance of need for school-based programs has been reflected in inclusion of life skill education program by NCERT and CBSC in present syllabus.

According to the UNESCO report 2008. (U.N.D.P. Report 2008) India stands at

- 102^nd^ position in the “Education for all developmental index” out of 129 countries
- 132^nd^ place in the list of 172 nations on human development index (HDI)

Ten per cent of 5-15 year old has a diagnosable mental health disorder. This suggests that around 50 million children under 18 would benefit from specialist services. There are up to 20 million adolescents with severe mental health disorders. Around 90% children with a mental health disorder are not currently receiving any specialist service.[4]

## REVIEW OF LITERATURE

### Attention deficit hyperactivity disorder

Prevalence of hyperactive syndrome in 2160 primary school children between 6-12 years was found to be 4.67%. Ratio of male to female distribution was 4.74:1. Sleep related problems may have significant bearing on the course and management of ADHD. At least one sleep related problem was present in 65.62% of children in ADHD group and 30% in the sibling group. A careful evaluation of sleep history is recommended in children with ADHD. ADHD is observed as a comorbid psychopathology in large number of disorders like substance abuse, mental retardation, autism, conduct disorder, cerebral palsy, learning disorders and medical illnesses.[5,6]

### Child and family

A child is born and brought up in a family. Family dynamics plays a vital role in mental health and illness. Psychologically and physically broken home has been reported in both the depressive and schizophrenic psychopathology. Child rearing practices can retard or accelerate development of child health. Schizophrenogenic parents and refrigerator parents who are cold and apathetic, produce autistic and psychotic child behavior.[7–10]

### Co morbid disorders

Prevalence of behavioral disturbance in children with nephrotic syndrome was 68%, significantly higher than that in the control group 21.6%. The behavior abnormalities found in the nephritic syndrome group were hyperkinesis, obsessive compulsive neurosis, conduct disorder, and emotional disorder in that order. Frequency of relapse and low socio-economic status showed significant association with presence of behavior disorder in the nephritic syndrome group. This association persisted even after adjusting for other socio-demographic, disease, treatment related variables including steroid therapy.[11]

### Delinquency, conduct problems and criminality

The evidence is clear that prevention and intervention must begin early, preferably during preschool years. Early intervention is especially critical for children growing up. Research strongly indicates that interventions becomes more difficult and encounters more intransigent behavior pattern from teenagers who exhibit antisocial behavior from an early age. Life course persistent offender who enters adolescence fully engaged in delinquent or antisocial behavior is usually highly resistant to change.[12]

The prevalence of the conduct disorder was 4.58% more common in boys, majority had childhood onset and one-third had comorbid ADHD. A study of Esser and colleagues reported prevalence of 0.9%, while Kashani *et al*. reported prevalence of 8.7%; DSM IV reports prevalence in males 6-10% and females 2-9%. The ratio of male to female CD is lower for the adolescent onset type than for the childhood onset type. Among the Indian studies Deivasigamani has reported the prevalence of CD to be 11.13%, Sarkar *et al*. reported prevalence rate of antisocial behavior to be 7.1% while recently Srinath *et al*. have reported prevalence as low as 0.2%. Attention-deficit hyperactivity disorder (ADHD) is the common co morbidity in children with conduct disorder.[13]

### Epidemiology

Various studies have shown a higher prevalence of different specific developmental disorders in males, being two to four times more common in boys than girls. Malhotra *et al*. while studying the incidence of childhood psychiatric disorders in the community found its rate to be 18/1000 years; the rate could be said to range between18-37/1000/years. The highlighted the need for such large scale studies to understand the rates and pattern of causation.[14,15]

### Mental retardation

Considerable focus has been on study of various facets of mental retardation. The area that has been discussed widely include types of retardation, assessment, prevalence, causes, genetic and biochemical screening, study of behavioral patterns, comorbidity personality pattern of parents and their attitude along with socio-economic studies. Fewer studies have focused on multidisciplinary management and long term care in institution for children with mental retardation.[16–20]

### Neurosis

Neurotic disorders constitute one of the common psychiatric problems met with in children. Among the neurotic disorders majority of the cases reported were hysteria (40-70%). Children tend to pick up considerable symptom pathology from parents or within the family.[21–24]

### Psychopharmacology

Psychiatric illnesses are common during pregnancy and post-partum period. Treatment of this condition is important as they can affect the health of the mother and fetus/infant. Issues become even more complex with multiple drugs and if there are associated medical problems or the infant is premature. Important issues to be kept in mind include folate supplementation in all women in the reproductive age group; planning pregnancies to minimize fetal exposure, discussion with the patient and family regarding options and documentation of all discussions and decisions. Active liaison with obstetricians, ultrasonologist and pediatricians need to be stressed, as also the need for local registries. Whenever possible use non-pharmacological approaches in addition. If psychotropic is necessary the choice of medication should be guided primarily by its safety data during pregnancy and breast feeding and by the psychiatric history of the patient.[25]

Children are not miniature adults. Psychopharmacokinetics and dynamics needs to be understood before administration of any molecule. One needs to know longitudinal safety data on child development and growth.

### Schizophrenia

Schizophrenia has been considered to be a neuro developmental disorder and the Childhood Onset Schizophrenia (COS) offers an important opportunity to examine abnormal neurodevelopment in this disorder. SPECT and neuropsychological findings in COS converge with those of Adult Onset Schizophrenia (AOS) but with greater temporal lobe involvement in COS. This is the first SPECT study in COS; therefore, the findings could be compared only with MRI studies that are available and depicts structural abnormalities. There is complex interaction between structural and functional systems in the CNS. As SPECT studies advance it may be possible to physiologically characterize, classify and diagnose various neurobiological and psychiatric disorders and possibly predict therapeutic effect and outcome.[26]

### Substance abuse

Rajkumar Bansal’s study highlights substance use pattern of 300 child laborers from six slums in Surat city and identifies the microsocial and macro-social stressors which initiates and perpetuates their substance use. It was observed that 135 (45%) of the child laborers had used some substance with mean 1.5 substances used per child. Tobacco smoking was the most common form of substance abuse, followed by tobacco chewing, snuff, cannabis and opium. This study highlights an urgent need for the containment of substance abuse by these vulnerable early initiators. The ‘maturation hypothesis’ that young people grow out of their drug related problems as they grow older need to be revised. India has 15-45 million child laborers at risk who are subject to rampant and unabated exploitation.[27–30]

Significantly higher number of early onset alcohol dependence subjects had history of ADHD and/or ADD, RT compared to the late onset. There is need for greater evaluation of ADHD in populations of adults with alcohol dependence, especially those with early onset, and more intensive management of this high risk group in view of their poorer prognosis. Since treatment of ADHD in adolescent including stimulants is known to reduce substance use, including alcohol use, assessment of comorbid ADD, RT in adults has important therapeutic implications.[31]

### Suicide

Sharma *et al*. reported about 15.8% adolescents in South Delhi thought of attempting suicide while 5.1% actually attempted suicide. Females had both thoughts and attempts higher as compared to males. Sudhir Kumar has reported increased incidence of adolescent suicide world over and its relation to stressful life event in the recent past. Senseman reported schizophrenia as most common psychopathology in adolescents attempting suicide. The establishment of a ‘Crisis Center’ or a suicide prevention center in the major cities of India should be an urgent undertaking. A telephone answering service manned by volunteer person 24 hours a day throughout the year would be of great value.[32,33]

## CASE REPORTS

Considerable number of interesting case reports has been published. Some of them are:

### Title of the study

Effectiveness of fluoxetine in the treatment of skin picking: Due to its similarities between OCD and impulse control disorder, skin-picking and related behaviors have been reported to span a compulsivity-impulsivity continuum from the purely obsessive- compulsive to the purely impulsive, with mixed symptoms in between. This case report highlights the effectiveness of fluoxetine in the treatment of skin-picking and the need for early institution of treatment, which would not only curtail the period of suffering but ensure long-lasting improvement in such cases.[34]

#### Management of trichotillomania

Treatment package of combination of pharmacotherapy and behavior therapy showed significant improvement. Various other studies explored insight oriented psychotherapy, cognitive-behavioral therapy but documented three studies in this report showed very encouraging results in managing this disorder of impulse control.[35]

#### Conduct disorder

A sequelae of viral encephalitis: Hyperactivity and oppositional behavior in children are common post encephalitis sequelae. Some of the great neuropsychiatrists of the past described the behavioral and temperamental changes that can occur following VE.[36]

#### Rett’s Syndrome

Increased identification will help in greater understanding of Rett’s Syndrome and proper guidance will help to reduce the burden of care on the parents.[37]

#### Clozapine in pregnancy

Like other psychotropics clozapine should be used with caution during pregnancy as there is insufficient knowledge regarding clozapine-induced agranulocytosis in fetus/neonate.[38]

#### Somnambulism

Diagnosis and treatment: Careful noting of case history and epilepsy is an important differential diagnosis. Treatment is considered when frequency of events is high, psychosocial complications or stressors are present or when events are violent and potentially injurious. A low-dose benzodiazepine is the drug of choice, although tricyclic antidepressants and trazodone may be beneficial as well. Behavioral management in the form of scheduled awakenings and a positive bedtime routine along with appropriate interventions are essential.[39]

#### A case of early infantile Autism

The case was diagnosed as early infantile autism with cerebral palsy and mental defect on the basis of the peculiar behavior and language, echolalia and mutism.[40]

#### A case of Kleine Levine Syndrome

The case reported fitted into the diagnostic criteria of Critchley and Hoffman (1942) with respect to age of onset and periodic attacks of excessive sleep and eating. Long term follow-up supported the diagnosis of it as the unmistakable case of Kleine Levine Syndrome.[41]

#### An unusual variant of Hallerman Streiff Syndrome

Case differed from the classical Hallerman Streiff in having no ophthalmological problems which is a common finding and in having chromosomal anomaly, but many other features like mandibulo-oculo-dyscephaly making it an unusual variant. Presence of Psychosis was also an uncommon occurrence. Possible emergence of new syndrome was considered.[42]

#### Treatment-refractory, juvenile-onset bipolar affective disorder

The cumulative incidence of bipolar disorder in childhood and adolescence may equal the 1% rate in adults. An 18-year-old girl diagnosed with bipolar affective disorder was given trials with various mood stabilizers as a monotherapy and combination therapy for current episode. Treatment resistant status led to initiation of clozapine with which patient stabilized within 4 weeks on 200 mgs. Patient maintained remission during next 2 years of follow-up. The natural history and management of juvenile bipolar disorder present more questions than answers.[43]

## CONCLUSIONS

It was not possible to cover all the topics and articles published in Indian Journal of Psychiatry on child and adolescent mental health in the last five decades. There are 118 original articles, 21 case reports, six editorials, five presidential addresses and one oration dealing with the subject. It is our humble suggestion to refer to large number of books written by Indian psychiatrist, psychologist and social workers on child and adolescent mental health. Apart from English, large number of publications on the area is in vernacular languages. It is advisable to search Indian research material from various other sources like IAPP Journal, IACAM Biannual reports, Indian Journal of Pediatrics and Indian Journal of Preventive and Social Work. Large numbers of services are primarily multidisciplinary; other team members publish their work in their respective association journals. There are specialized groups which function independently with their own association like Down’s syndrome Association, Spastic Society, Society for mentally handicap children, Dyslexia association and national forum for autism.
